# Biosynthesis and Application of Catechins and Their Derivatives in *Camellia sinensis*

**DOI:** 10.1002/fsn3.71277

**Published:** 2025-11-30

**Authors:** Haiyi Yao, Yuxin Gu, Dibin Zhu, Dandan Tang, Wei Chen, Yongxian Chen, Jiaojiao Zhang, Liqiang Tan

**Affiliations:** ^1^ Tea Resources Utilization and Quality Testing Key Laboratory of Sichuan Province Sichuan Agricultural University Chengdu China; ^2^ College of Horticulture Sichuan Agricultural University Chengdu China; ^3^ College of Food and Health Zhejiang A& F University Hangzhou China; ^4^ Biological Physics Group, University of Manchester Manchester UK

**Keywords:** biosynthesis, catechin derivatives, catechins, tea plant (*Camellia sinensis*), tissue‐specific accumulation

## Abstract

Tea is one of the world's most widely consumed non‐alcoholic beverages and is a rich source of bioactive compounds. The synthesis and accumulation of catechins, central flavonols in the secondary metabolism of the tea plant, not only define the bitterness and astringency of the tea infusion but also form the chemical cornerstone of its remarkable antioxidant activity, playing a key physiological role in the plant's abiotic stress tolerance. Catechin biosynthesis involves a complex enzymatic network in tea plants ( *Camellia sinensis* ). With the development of molecular biotechnology, the biosynthetic and chemical pathways have been progressively clarified, and the roles of key enzymes have been increasingly understood. Moreover, accumulating evidence shows that catechin distribution displays tissue‐specific patterns, providing important references for genetic improvement and utilization of tea resources. This review summarizes current knowledge on catechins and their derivatives, including biosynthesis and applications. It also highlights the specific accumulation of monomeric and polymeric catechins in different tissues and germplasm and discusses their prospective applications in functional foods, pharmaceuticals, and other health‐related industries. This work synthesizes the biosynthesis, tissue accumulation, and multifaceted health benefits of catechins, offering a theoretical basis for optimizing tea germplasm and deep‐processing. It underscores the nutrient and medicinal potential of catechins, thereby informing the development of functional foods and pharmaceuticals and enhancing the value chain of the tea industry.

## Graphical abstract

This review summarizes the biosynthesis, structural diversity, and functional properties of catechins in tea. It highlights their tissue‐specific accumulation in different tea tissues and discusses the potential applications of catechin derivatives in functional foods and health‐related products.

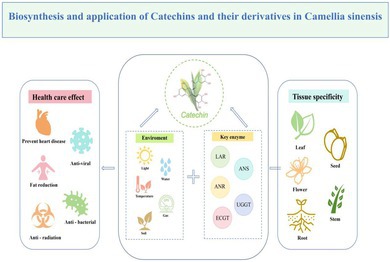

## 1 Introduction

As one of the world's three major non‐alcoholic beverages, tea owes much of its quality and health benefits to its main secondary metabolites, catechins. Since Fujiki et al. first reported the inhibitory effects of epigallocatechin gallate (EGCG) on human cancer cells in 1987 (Fujiki et al. 2002), numerous studies from countries such as the United States, Japan, China, the United Kingdom, and Italy have elucidated the antioxidant activities and mechanisms of catechins through thousands of in vivo and in vitro experiments, clinical trials, and epidemiological investigations. The metabolism and transformation of catechins within the tea plant, as well as the derivatives produced by artificial modification, have significantly expanded and optimized their original pharmacological activity profiles, and they may potentially be used as eco‐friendly stabilizers and functional additives (Latos‐Brozio et al. 2020). Recent advances in molecular and cell biology have increasingly revealed the health benefits of catechins and their derivatives through multidisciplinary research (Table 1). These benefits include antioxidant activity (Ghosh et al. 2024), antiviral effects (Xu et al. 2017), anti‐inflammatory properties (Suprahman et al. 2024), anti‐aging effects (Holghoomi et al. 2025), antihypertensive activity (Suprahman et al. 2024), radiation protection (Zhao et al. 2021), and lipid metabolism regulation (Liu et al. 2025). Current research on catechins and their derivatives focuses on elucidating their biosynthetic pathways, cloning and expressing functional genes, clarifying their bioactive mechanisms, and exploring applications in health foods, pharmaceuticals, and cosmetics.

**TABLE 1:** Pharmacological effects of catechins.

Pharmacological effect	Application	References
Antibacterial	Nanoformulations improve antibacterial activity by enabling controlled drug release and facilitating the enhanced bioavailability of polyphenols	Lui et al. (2023)
Prevent respiratory diseases	In vitro anaerobic fermentation was employed to investigate the microbial metabolism of GTCs by human fecal microbiota and its dynamic alteration, which led to enhanced antioxidant, α‐amylase inhibitory, and α‐glucosidase inhibitory activities in the fermented GTCs sample compared to the unfermented control	Su et al. (2024)
Anti‐inflammatory	The anti‐inflammatory and antioxidant properties of catechins—mediated through the suppression of inflammatory pathways/cytokines and the chelation of metal ions/scavenging of radicals, respectively—may also underlie their inhibitory effects on tau protein phosphorylation, amyloid‐beta aggregation, and the release of apoptotic proteins	Özduran et al. (2021)
Mitochondrial function protection	Through the mitigation of oxidative damage, catechins improve mitochondrial function and stimulate biogenesis. These actions collectively sustain neuronal energy metabolism by preserving mitochondrial integrity, thereby delaying the progression of disease	Li et al. (2023)
Prevent hypertension	Aimed at evaluating the antihypertensive effect of the natural polyphenol catechin (CAT) and its action on renal arachidonic acid (AA) metabolism in comparison to captopril (CAP), this study demonstrated that both low and high doses of CAT attenuated the development of hypertension in SHRs. Notably, the high‐dose group exhibited a significant blood pressure reduction relative to the untreated controls	Elbarbry et al. (2022)
Prevent heart disease	Catechin administration attenuated coronary heart disease (CHD) by normalizing serum biomarkers of cardiac injury (creatine kinase, CK‐MB, lactate dehydrogenase, and cTnT) and improving cardiac function parameters (LVEF and LVIDs)	Tu et al. (2018)
Fat reduction	Fecal metabolome analysis revealed that GTC treatment reduced saturated fatty acids and improved amino acid levels, thereby supporting gut health and metabolism. Furthermore, the study demonstrated that GTC prevents obesity‐induced renal damage by modulating PPARγ/CD36 signaling and maintaining gut homeostasis in rats	Patial et al. (2023)
Antiaging	Catechins exert antioxidant effects through a dual approach: acting as an exogenous scavenger of lipid peroxidation products, and activating the endogenous antioxidant system by modulating enzyme activities and signaling pathways. These actions contribute to their potential in the prevention and treatment of atherosclerosis (AS)	Sheng et al. (2023)
Oral care	By inhibiting the growth and metabolism of oral bacteria, catechins reduce dental plaque formation and acid production, thus achieving caries prevention	Malcangi et al. (2023)
Antiradiation	Correspondingly, EC mitigated apoptosis in intestinal crypt cells following radiation. This protection was triggered by EC promoting the translocation of Nrf2 from the cytoplasm to the nucleus, a process that initiated the upregulation of HO1 and NQO1 expression	Li et al. (2019)
Antibacterial	The synthesized ZnO/catechin nanocomposite demonstrates a potent antibacterial effect. Notably, it exhibits a significantly lower minimum inhibitory concentration than its individual components and outperforms azithromycin, validating its superior antibacterial potential	Kader et al. (2024)
Prevent depression	(−)‐EGCG exerts its significant antidepressant effects via concerted activation of the AMPK‐mTOR autophagy axis, inhibition of the NLRP3 inflammasome, and enhancement of BDNF expression, thereby ameliorating both behavioral and biochemical abnormalities in CUMS mice	Zhang et al. (2023)

The accumulation of catechins and their derivatives in fresh leaves is a complex process influenced by gene expression, environmental conditions, developmental stages, and metabolic pathways in tea plants. Although the biosynthetic pathway of catechins has been largely elucidated and most of the genes involved in their synthesis and regulation have been cloned and studied (Jiang et al. 2025), the precursors for catechin biosynthesis are mainly provided by the shikimate and phenylpropanoid metabolic pathways, followed by the generation of various catechin monomers with the participation of a series of enzymes (Zhao et al. 2020). However, due to the complexity of gene regulation and the dual impact of environmental factors, the accumulation of different catechin monomers in tea plants exhibits significant specificity. Some researchers have found that EGCG and gallocatechin gallate (GCG) can be interconverted in tea plants (Jiao et al. 2023), and this dynamic equilibrium may be closely related to the metabolic regulatory mechanisms of tea plants at different growth stages or under environmental stress. Moreover, catechin derivatives are extremely low in natural systems. Therefore, systematically elucidating the biosynthetic pathways of catechins and their derivatives is of crucial strategic significance. In‐depth analysis of the biosynthetic pathways of catechins and their derivatives will not only help to unravel the complexity of the secondary metabolic network in tea plants but also provide a theoretical basis for tea quality regulation, molecular breeding, and innovative applications. Recent studies have demonstrated that the autoxidation of epicatechin (EC) and epicatechin gallate (ECG) can lead to browning phenomena. This finding not only reveals the chemical reactivity of these flavan‐3‐ols but also suggests their potential role as building units for proanthocyanidin polymerization, which holds significant implications for both the biosynthetic pathways and stability of these compounds (Tan, Li, et al. 2023; Tan, Vincken, et al. 2023). Meanwhile, a colorimetric sensor array (CSA) technology incorporating nanozyme peroxidase (POD)‐like activity has been successfully applied for the efficient discrimination of polyphenols in tea. This innovative approach offers a new strategy for the accurate assessment of tea quality and the detection of adulteration, further underscoring the necessity of in‐depth investigations into the biosynthetic pathways of catechins and their derivatives (Tan, Li, et al. 2023; Tan, Vincken, et al. 2023).

Although the general biosynthetic pathway of catechins has been previously outlined, a systematic discussion of the gene regulatory networks fine‐tuning this pathway remains fragmented, offering only a partial depiction of catechin production. A comprehensive mechanistic understanding of the tissue‐specific accumulation of catechins in *Camellia sinensis* and its modulation by environmental factors is still conspicuously absent. Future research should prioritize the use of multi‐omics technologies to fully decipher the catechin metabolic regulatory network, develop molecular breeding strategies for precise manipulation of catechin content, and expand their applications in functional foods and pharmaceuticals.

This review synthesizes recent advances in catechin biosynthesis and the formation of diverse derivatives, with a dedicated focus on the roles of key biosynthetic enzymes. Distinctively, it elucidates the unique mechanisms governing the tissue‐specific accumulation of these compounds in *Camellia sinensis* —a critical determinant of tea quality. By bridging these molecular insights with strategies for metabolic engineering and industrial application, this work provides a fresh perspective that transcends conventional summaries. It thereby connects fundamental plant science with tea industry innovation, offering a scientific foundation for optimizing tea germplasm and developing catechin‐based products.

## 2 Catechin Derivatives and Their Biosynthesis

Research on catechins and their three main bioactive derivatives—catechin oxidation polymers, methylated catechins, and catechin‐theanine conjugates—has progressed from initial activity characterization to more advanced stages aimed at elucidating structure–activity relationships and achieving precise synthesis, with much of this progress deeply rooted in studies of the tea plant ( *Camellia sinensis* ). Similarly, the exploration of catechin biosynthesis has evolved from the cloning and functional verification of key enzyme genes in tea to the in‐depth dissection of multi‐layered regulatory networks and efforts toward synthetic biological reconstruction. However, several scientific questions specific to *Camellia sinensis* remain to be systematically clarified, including the regulatory mechanisms of transcription factors, the allocation of metabolic flux between different branches, the interconversion of isomers, and the impact of environmental factors on this flux. Therefore, a precise dissection of the biosynthetic pathways of catechins and their derivatives in tea is not only of profound theoretical value—revealing the evolutionary strategies and regulatory logic of plant secondary metabolism in a key crop—but also possesses significant application potential. Breaking through the bottleneck in pathway elucidation represents a critical turning point for catechin production, shifting it from traditional extraction from tea leaves toward rational design and manufacturing. This paradigm shift will profoundly accelerate innovative applications of catechins derived from *Camellia sinensis* in pharmaceuticals, nutrition, and functional materials.

### 2.1 Catechin Derivatives

Catechins (flavan‐3‐ols), the primary bioactive components in tea (Chen et al. 2020), account for 14%–24% of the dry weight of fresh tea leaves (Li et al. 2023). There are eight monomeric catechins: EC, epigallocatechin EGC, ECG, EGCG, catechin, gallocatechin (GC), catechin gallate (CG), and GCG. Among these, EGCG is the most abundant and potent catechin, accounting for over 50% of the extractable substances in green tea (Zhao et al. 2020; Zhang et al. 2025). Its low redox potential and multiple ortho‐phenolic hydroxyl groups enable it to donate hydrogen atoms to interrupt or terminate free radical chain reactions, a fundamental structure that confers its strong free radical scavenging ability (Zhang et al. 2025). As the main functional components in tea, catechins possess significant biological activities and health benefits. However, the bioactivity of catechins is not static. It can be transformed through the tea plant's own metabolic processes or modified artificially, which can greatly expand and optimize their original pharmacological activity profile (Wu and Brown 2021). Current research indicates that catechin derivatives produced by electrophilic substitution reactions during tea plant growth or processing have garnered attention due to their remarkable antioxidant, anti‐inflammatory, metabolic regulatory, and neuroprotective activities (Wang et al. 2025). However, these highly active derivatives are extremely low in natural systems, and their chemical synthesis faces significant challenges such as difficulties in controlling regio‐ and stereoselectivity, low efficiency, and complex environmental conditions, which severely restrict in‐depth functional studies and industrial applications.

The B‐ring phenolic hydroxyl groups of catechins are prone to oxidation, forming ortho‐quinones, which are highly unstable and readily undergo complex polymerization and condensation reactions under the catalysis of polyphenol oxidase (PPO), leading to the formation of ortho‐quinone derivatives and further polymerization into dimers and polymers. Mitochondrial activation factors (MAFs) can increase mitochondrial membrane potential (Kuban‐Jankowska et al. 2020). Subsequent studies have shown that MAFs are also oxidation and polymerization products of catechins, possibly generated by further oxidation and polymerization of theaflavins or ester‐type catechins (He et al. 2023). These phenolic compounds, due to their unique structure, possess strong antioxidant capabilities and can improve mitochondrial function, delay aging, and regulate immune metabolism, similar to resveratrol, coenzyme Q10 complex, and nicotinamide riboside (NR). Methylated catechins are methylated derivatives of the catechin EGCG, with primary methylation sites being the 3′ or 4′ hydroxyl groups on the B‐ring (such as EGCG3″Me and EGCG4″Me). The replacement of phenolic hydroxyl groups with methoxy groups enhances their lipophilicity and metabolic stability, as well as their antioxidant, anti‐inflammatory, and anticancer activities, with particularly significant antiallergic effects (Zhuang et al. 2024). The earliest discovery of eight N‐ethyl‐2‐pyrrolidinone‐substituted compounds (N‐ethyl‐2‐pyrrolidinone‐substituted flavan‐3‐ol, EPSF) in dark tea was by Wang et al. These compounds are formed by the reaction of catechin components (i.e., EGCG, ECG, EGC, EC, catechin, GC, etc.) in tea with free amino acids like theanine, involving N‐ethyl‐2‐pyrrolidinone substitution at the C‐8 or C‐6 position of catechins (Wang et al. 2014). As natural condensation products of catechins and theanine, EPSFs exhibit significant advantages in neuroprotection and vascular disease intervention through multi‐target actions.

### 2.2 Biosynthesis of Catechins

**FIGURE 1:**
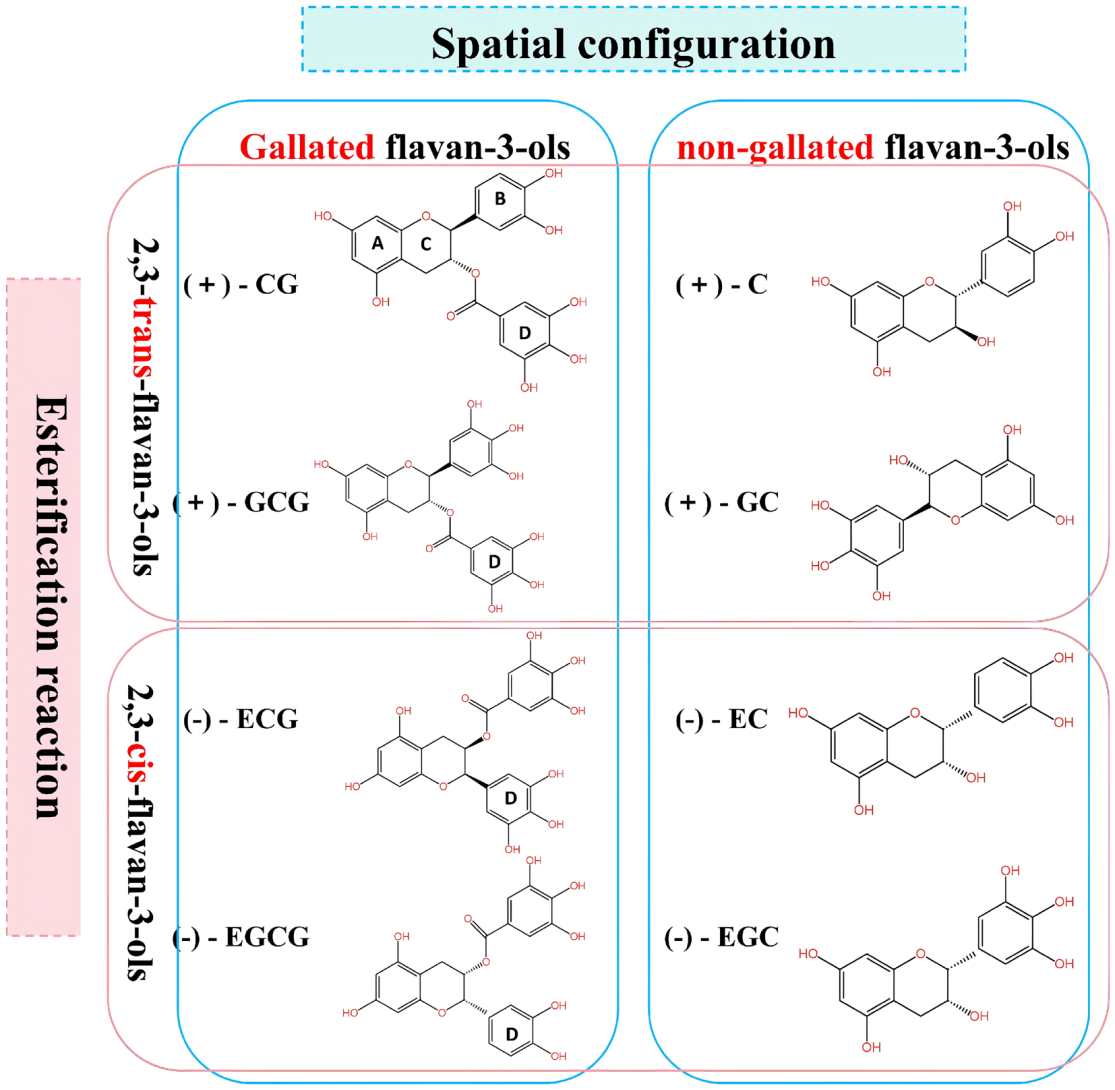
Two classification methods of catechins. In the structure, rings A–D represent distinct carbocyclic moieties, with ring D denoting the galloyl group characteristic of ester‐type catechins. The notation “(+)” indicates trans‐catechins, while “(−)” denotes cis‐catechins. The abbreviations and full names of various catechins are as follows: C, catechin; CG, catechin gallate; EC, epicatechin; ECG, epicatechin gallate; EGC, epigallocatechin; EGCG, epigallocatechin gallate; GC, gallocatechin; GCG, gallocatechin gallate.

Catechins are derivatives of 2‐phenylbenzopyran and belong to the flavan‐3‐ol subclass of flavonoids, representing an important constituent in tea leaves. There are primarily eight monomeric catechin compounds, which can be categorized in two main ways (Figure 1). First, based on the spatial orientation of the substituents at positions C2 and C3 on the pyran ring, they are divided into ECs (*cis*‐catechins, including EC, EGC, ECG, and EGCG) and catechins (trans‐catechins, including catechin, GC, CG, and GCG). Second, depending on whether the hydroxyl groups on the C ring of catechins undergo esterification with gallic acid (GA), they are classified as esterified catechins (CG, ECG, GCG, and EGCG) or non‐esterified catechins (catechin, GC, EC, and EGC) (Zhu et al. 2020).

**FIGURE 2:**
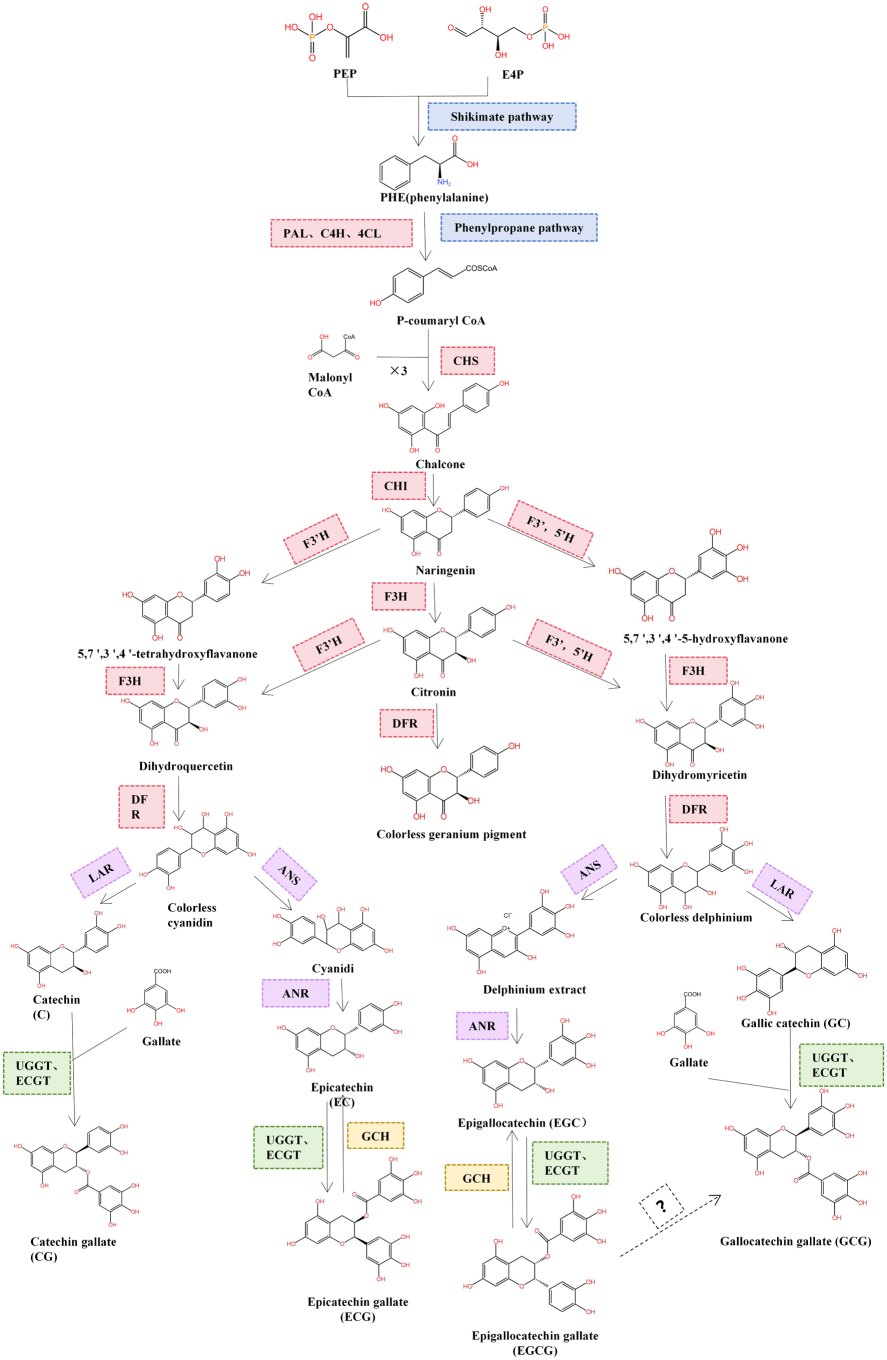
The biosynthetic pathway of catechins. In the biosynthetic pathway of catechins, leucoanthocyanidin reductase (LAR), anthocyanidin synthase (ANS), and anthocyanidin reductase (ANR) play pivotal roles in the synthesis of non‐esterified catechins; UDP‐glucose: Flavonoid‐3‐O‐glucosyltransferase (UGGT) and catechin gallate transferase (ECGT) serve as key enzymes in the synthesis of esterified catechins.

The biosynthetic pathways of flavonoids have been elucidated (Figure 2). However, due to the complexity of polyphenolic compounds, the biosynthetic pathways constitute a complex and intricate regulatory network. The upstream enzymes involved in phenylpropanoid metabolism and flavonoid synthesis pathways exhibit strong conservation and evolve more slowly compared to downstream enzymes. The biosynthesis of catechins in tea plants mainly consists of four parts: the shikimic acid pathway, the phenylpropanoid pathway, the synthesis of non‐gallate catechins, and the synthesis of gallate catechins.

In tea plants, the biosynthesis of catechins begins with two basal metabolic pathways. In the shikimate pathway, erythrose 4‐phosphate (E4P) from the pentose phosphate pathway and phosphoenolpyruvate (PEP) from glycolysis form 3‐deoxy‐D‐arabino‐heptulosonate 7‐phosphate (DAH7P) via 3‐deoxy‐D‐arabino‐heptulosonate 7‐phosphate synthase (DAHPS). DAH7P then undergoes a series of enzyme‐catalyzed reactions to yield aromatic amino acids, vitamins, lignin, phenolic compounds, and alkaloids, providing precursors for subsequent approaches (Tang et al. 2022). Phenylalanine is converted into p‐coumaryl‐CoA by phenylalanine aminolase (PAL) and enters the phenylalanine pathway, eventually generating p‐coumaroyl‐CoA. Subsequently one molecule of p‐coumaroyl‐CoA and three molecules of malonyl‐CoA react under chalcone synthase (CHS) to form naringenin chalcone, which is cyclized to naringenin by chalcone isomerase (CHI). Chalcones are key precursors for catechins and anthocyanins, forming their basic molecular skeleton. The O1–C2 linkage creating a pyran ring offers crucial insights for catechin chemical synthesis. Naringenin undergoes hydroxylation at the B‐ring by flavonoid 3′‐hydroxylase (F3′H) and flavonoid 3′,5′‐hydroxylase (F3′,5′H) to form flavanones, which are then reduced to leucoanthocyanidins by flavonoid 3‐hydroxylase (F3H) and dihydroflavonol 4‐reductase (DFR).

Starting from the common precursor leucoanthocyanidins, the biosynthetic pathway of catechins differentiates into two main branches. Leucoanthocyanidins can be directly reduced by leucoanthocyanidin reductase (LAR) to non‐epimeric catechins, catechin and GC (Jiang et al. 2025), they can also be sequentially catalyzed by anthocyanidin synthase (ANS) and anthocyanidin reductase (ANR) to form the epimeric catechins, EC and EGC (Liu et al. 2016). Isotopic labeling experiments indicate possible EC/EGC interconversion.

In tea plants, the accumulation of catechins is predominantly composed of ester‐type catechins, which account for 70% of the total catechin content. Therefore, the galloylation of catechins is a crucial step in the accumulation of catechins in tea plants. Researchers consider 1‐O‐galloyl‐β‐glucose an effective acyl donor and receptor in tannin synthesis (Montes‐Ávila et al. 2017). Jiang et al. proposed the existence of a “galloylation–degalloylation cycle (G‐DG)” pathway in tannin‐rich plants. This pathway, consisting of three catalytic steps controlled by gallic acid glucosyltransferase (UGT84A22), serine carboxypeptidase‐like acyltransferase (SCPL‐AT), and tannase (TA), is not only the main terminal pathway for the biosynthesis of galloylated catechins but also the primary initial pathway for the biosynthesis of hydrolyzable tannins (Jiang et al. 2025). The catechin galloylation process is analogous to the hydrolyzable tannin biosynthetic pathway, where 1‐O‐galloyl‐β‐glucose directly donates acyl groups for the synthesis of ester‐type catechins. Under the enzymatic actions of UDP‐glucose: flavonoid‐3‐O‐glucosyltransferase (UGGT) and catechin gallate transferase (ECGT), EC and EGC form ester‐type catechins (Liu et al. 2012). Qian et al. (2015) demonstrated, via chiral‐phase HPLC, that a partially purified native galloylated catechin hydrolase (GCH) from tea ( *Camellia sinensis* ) catalyzes the hydrolysis of ECG to release EC, providing direct evidence for GCH‐mediated degalloylation in catechin metabolism (Qian et al. 2015). Recently, studies have confirmed that the enzyme involved in the degalloylation of catechins belongs to the hydrolase superfamily, specifically the carboxylesterase class of tannases (Dai, Liu, et al. 2020; Dai, Lou, et al. 2020). The galloylation and degalloylation processes catalyzed by TA can occur simultaneously in plants (Chen et al. 2023). *CsTA* can function as both a hydrolase and an acyltransferase, catalyzing galloylation and degalloylation, respectively.

Although the various catechin monomers are generated in tea plants through the catalysis of different enzymes, their contents and ratios are not fixed. Under certain conditions, interconversion between them can occur. In vitro enzyme activity assays have demonstrated that laccase can catalyze the oxidation of EGCG to produce simple catechins, thearubigins, and small amounts of ester‐type catechins (Lin et al. 2015). However, the mechanism of conversion between EGCG and GCG remains to be further explored.

### 2.3 Synthetic Pathways of Catechins Derivatives

Catechin derivatives, through structural modifications, can significantly enhance their pharmacological activity and bioavailability, thereby improving their absorption and distribution in the body. These modifications can also extend the half‐life of catechins in the blood and strengthen their biological activities, such as antioxidant, anti‐inflammatory, and antitumor effects (Table 2). Catechin derivatives demonstrate tremendous potential in drug development, and this strategy provides a crucial pathway for developing more efficient and safer catechin‐based drugs.

**TABLE 2:** The functions and applications of catechin derivatives.

Bioactive combination	Functions and applications	References
MAFs + HIF‐1α	By modulating HIF‐1α stability in fibroblasts to enhance hypoxic adaptation, it offers a promising nutritional strategy for managing ischemic diseases and altitude sickness	Choya‐Foces et al. (2024)
MAFs + nanocarrier system	A promising strategy involves enhancing the bioavailability and targeting of MAFs via liposomal or polymeric nanocarriers, thereby developing efficient delivery systems to improve their therapeutic efficacy in specific tissues such as muscle or brain	Mita et al. (2022)
MAFs + caffeine	This compound boosts antioxidant and cytoprotective activity by stabilizing cell membranes and suppressing oxidative stress, reducing cell damage. This makes it ideal for sports supplements and functional beverages to enhance endurance and minimize exercise‐induced injury	Das et al. (2020) and Yashima et al. (2021)
MAFs + collagen fibers	The stability and recovery of MAFs are enhanced through hydrogen bonding and hydrophobic interactions, thereby minimizing activity loss during processing. This strategy is employed in tea‐extract purification to efficiently isolate catechin compounds while preserving their bioactivity	Yashima et al. (2021) and Zhang et al. (2023)
MAFs + dietary fiber (e.g., cellulose)	MAF bioaccessibility is modulated via adsorption, with pH‐dependent binding governing intestinal release and absorption—a mechanism exploited in gut‐health products for sustained polyphenol release and enhanced colonic antioxidant effects	Jakobek et al. (2018)
Theaflavins + hydrogen sulfide modulators	Through H_2_S‐mediated sulfhydration, the activation of SOD and GSH counteracts oxidative damage, a process that contributes to delayed vascular aging and the mitigation of associated degenerative diseases.	(Li et al. 2019)
EGCG4′′Me + nano‐PLGA carrier	Nano‐encapsulation enhances the bioavailability and stability of methylated EGCG, thereby improving its cellular uptake efficiency. This enhanced delivery platform can be utilized in targeted systems to boost the efficacy of cancer chemotherapy, particularly against challenging cases such as breast cancer and multidrug‐resistant tumors	Wong et al. (2021)
EGCG4′′Me + soy protein isolate	It protects MAFs from degradation in acidic environments, thereby enhancing their intestinal absorption and anti‐inflammatory efficacy, particularly against metabolic disorders. This protective strategy allows for its application in plant‐based functional foods aimed at cardiovascular health management and weight control	Miyawaki et al. (2018)
EGCG4′′Me +β‐glucan	Through the dual modulation of hepatic lipid genes and cholesterol excretion, it synergistically reduces both LDL‐C and ox‐LDL levels, making it a suitable active ingredient for cholesterol‐lowering functional foods and beverages aimed at supporting cardiovascular health	Suzuki et al. (2013, 2000)
EGCG3′′Me + collagen peptides	It synergistically inhibits matrix metalloproteinase‐1 (MMP‐1) and oxidative stress while enhancing dermal fibroblast activity, thereby reducing wrinkles and signs of photo‐aging. These effects support its application in beauty nutraceuticals designed for anti‐aging and skin moisturization	Hill et al. (2007)

Monomeric catechins (such as EGCG and ECG) serve as precursors to MAFs. These monomeric substances undergo catalytic oxidation by PPO or POD to form dimers to polymers. The first step is initial oxidation, the generation of quinone intermediates, which can be accomplished through two pathways. One is the PPO pathway, where the catechin B‐ring catechol hydroxyl groups are oxidized to ortho‐quinones by PPO, such as EGCG quinone (Abudureheman et al. 2022). The other is the POD pathway, where in the presence of H_2_O_2_, POD catalyzes the oxidation of EGCG, simultaneously producing H_2_O_2_ as a byproduct. The second step is dimerization, leading to the formation of theaflavins (TFs). Catechin quinones condense with nucleophilic sites of unoxidized catechins. For example, EC quinone condenses with EGC to form theaflavin TF, and EGCG quinone condenses with ECG to form theaflavin‐3,3′‐digallate (TF3) and so on. The third step is higher‐order polymerization. TFs are further oxidized to generate phenanthraquinone rings (such as theaflavic acid) or ring‐opening products, which are oxidized to thearubigins (orange‐red polymers) under the action of POD/H_2_O_2_, eventually forming theabrownins. Subsequently, quinones spontaneously polymerize to form complex polymers with a molecular weight > 1 kDa. The final stage is mitochondrial activation. TFs enhance mitochondrial bioactivity by activating the AMPK‐PGC‐1α pathway and are representative components of MAFs (Abudureheman et al. 2022). This synthetic pathway is mainly influenced by pH, oxygen concentration, and differences in enzyme sources. At pH 4.5, POD activity is reduced, decreasing the conversion of TFs to thearubigins, with TF accumulation increasing by 40% (Misra et al. 2016). Anaerobic fermentation promotes TF retention, while high oxygen conditions accelerate polymerization (Li, Wen, Lai, et al. 2024; Li, Zhang, Li, et al. 2024; Li, Borg, Krammer, et al. 2024; Li, Zhang, Cui, et al. 2024). The enzymatic apparatus of loquat ( *Eriobotrya japonica* ) leaves, especially PPO and POD, efficiently oxidizes catechins and markedly accelerates the formation of theaflavin gallates and thearubigins. Co‐rolling loquat leaves with green tea elevates TF levels above those observed with tea leaves alone, indicating a higher oxidative capacity of the loquat enzymatic system (Tanaka et al. 2002).

Methylated catechins are derived from ester‐type catechins (such as EGCG), with S‐adenosylmethionine (SAM) serving as the methyl donor. These reactions are catalyzed by O‐methyltransferases (OMTs) to produce methylated catechins and S‐adenosylhomocysteine. Plant O‐methyltransferases can be divided into two classes: Class I, represented by caffeoyl‐CoA O‐methyltransferase; and Class II, represented by caffeic acid O‐methyltransferase (Xu et al. 2022). However, Class II enzymes lack O‐methyltransferase activity (Xu et al. 2022). To date, a type I catechol O‐methyltransferase (*CsOMT*) (Kirita et al. 2015) and a caffeoyl‐CoA O‐methyltransferase (*CCoAOMT*) gene (Zhang et al. 2015) have been cloned from tea plants. In vitro enzyme‐activity assays have confirmed their successful synthesis of various methylated catechins, including EGCG3″Me, EGCG4″Me, EGCG3″,5″‐diMe, EGCG3′,4″,5″‐triMe, and EGCG4″Me, EGCG3″Me, and EGCG3′Me. Jin et al. cloned *CsFAOMT1* and using the same primers, also cloned *CsFAOMT2*. They demonstrated that *CsFAOMT1* primarily catalyzes the synthesis of EGCG3″Me, while *CsFAOMT2* is closely associated with the synthesis of EGCG4″Me (Jin et al. 2023).

EPSFs are derivatives of catechins (EGCG/ECG) where the C‐6 or C‐8 position of the A‐ring is substituted by a nitrogen‐containing five‐membered ring (pyrrolidinone), and they exist as R/S enantiomers (e.g., S‐EGCG‐cThea, R‐EGCG‐cThea), with catechins and theanine as precursor substances (Gao et al. 2025). The biosynthetic pathways of EPSFs are multifaceted. One pathway is the enzymatic synthesis of basic EPSFs from fresh leaves: the electrophilic carbon (C‐6/C‐8) of the catechin B‐ring attacks the free amino group of theanine, resulting in dehydration condensation to form a pyrrolidinone ring. It is speculated that an endogenous condensing enzyme in tea plants (similar to an acyltransferase) is the key transferase for this process. Another pathway is the microbial transformation during dark tea post‐fermentation: during heap fermentation, Aspergillus and Penicillium genera secrete extracellular enzymes (PPOs, hydrolases) that catalyze condensation reactions, while microbial metabolites (quinones) act as electron acceptors to promote catechin oxidation and ring‐opening, enhancing reaction activity. There is also the non‐enzymatic reaction via heat treatment (Strecker degradation pathway): high‐temperature baking (such as the firing of oolong tea or the pan‐firing of yellow tea) promotes the oxidation and decarboxylation of theanine to produce acetaldehyde, which undergoes Strecker degradation to form the reactive intermediate 1‐ethyl‐5‐hydroxy‐2‐pyrrolidinone (EHP). The nucleophilic site of EHP attacks the catechin A‐ring to complete condensation (Gao et al. 2025).

## 3 Key Enzymes in Catechin Biosynthesis

At present, it has become a new research hot spot to use molecular biological research methods to reveal the secondary metabolic pathways of tea plants and their regulatory mechanisms. The catechin synthesis metabolic pathway is regulated by a variety of transcription factors. The key enzymes of catechin synthesis are located at the end of the flavonoid metabolic pathway. Generally, it is considered that DFR catalyzes the generation of colorless anthocyanins from dihydroflavonols. Then, under the action of LAR, ANS, and ANR, colorless anthocyanidins are transformed into catechins and ECs. Subsequently, after being acted upon by ECGT and UGGT, catechins and ECs are esterified into esterified catechins. The research progress of the above five enzymes will be discussed in this paper.

### 3.1 Leucoanthocyanidin Reductase (LAR)

Chen et al. (2022) reported that overexpression of LAR significantly enhances tannin biosynthesis, thereby providing indirect but compelling evidence for the catalytic role of LAR in the flavonoid pathway. In vitro reconstitution assays conducted by Jun et al. demonstrated that recombinant LAR and ANR enzymes catalyze the conversion of 3,4‐*cis*‐leucoanthocyanidin to catechin; however, the reaction efficiency is strongly modulated by pH, cofactor availability (especially NADPH), and the purity of the leucoanthocyanidin substrate (Jun et al. 2021). They detected DFR and LAR activities in crude tea plant enzymes for the first time. Using dihydroflavonols (DHK, DHQ, DHM) as substrates, the crude enzyme reaction generated products like afzelechin, catechin, and GC. This indirectly confirmed the dual function of DFR/LAR in tea plants. There are three key flavonoid pathway genes: ANS, ANR, and LAR. In tea ( *Camellia sinensis* ), Yang, Zhang, et al. (2024) and Yang, Li, et al. (2024) demonstrated that although overexpression of LAR can promote catechin biosynthesis, its effectiveness is limited by species‐specific metabolic channeling, resulting in undetectable levels of free catechins under certain physiological conditions (Yang, Zhang, et al. 2024; Yang, Li, et al. 2024). Further characterization of a tea *CsLAR* gene by Pang et al. (2013) revealed that the recombinant enzyme obtained from prokaryotic expression could catalyze the conversion of leucoanthocyanidin to (+)‐catechin in vitro. However, when *CsLAR* was overexpressed in tobacco, it led to an abundant accumulation of EC but only trace amounts of catechin (Pang et al. 2013).

*CsLARs* exhibit divergent catalytic functions and substrate preferences in vitro versus in vivo, revealing a complex physiological role. While in vitro assays typically demonstrate its ability to directly reduce leucoanthocyanidins to produce non‐ECs, in vivo studies have uncovered functionalities extending far beyond this canonical role. In 2016, Dixon's team found a new *MtLAR* substrate, 4β‐(S‐cysteinyl)‐EC, in alfalfa. *MtLAR* catalyzes the conversion of 4β‐(S‐cysteinyl)‐EC to EC, indicating a potential additional substrate for LAR in plants (Liu et al. 2016). Subsequent studies on the synthesis of proanthocyanidins in grapes further support this view that grapes also accumulate 4β‐ (S‐cysteine) ‐EC and 4β‐ (S‐cysteine) ‐C. *VvLAR1* and *VvLAR2* can catalyze not only leucoanthocyanidins but also these compounds to produce EC and C (Yu et al. 2019). These findings reveal a critical regulatory mechanism: the catalytic product profile of LAR is fundamentally governed by the identity of its accessible substrates. Previous studies indicate that LAR can participate in both epicatechin and non‐epi‐catechin synthesis. When the LAR substrate is C‐type leucocyanidin, C‐type catechins are produced. If the substrate is EC‐type leucocyanidin, the product is EC‐type catechins.

### 3.2 Anthocyanidin Reductase (ANR)

Proanthocyanidins are widely present in plants, and their biosynthetic mechanism has long been a challenging issue in the plant flavonoid metabolic pathway. The search for the terminal synthase in proanthocyanidin biosynthesis is ongoing. Correlation analyses have shown that in many plants, *ANR* gene expression is highly positively correlated with proanthocyanidin accumulation (He et al. 2024; Samynathan et al. 2023). Research on ANR can be traced back to 1997, when Albert et al. (1997) found a mutant in *Arabidopsis thaliana* . They named it Banyuls (BAN) as its seeds accumulated red substance on the seed coat surface before maturation, resembling the color of Banyuls wine. In 2003, Xie et al. from Dixon's team found a homologous sequence of the BAN gene in alfalfa. When BAN from alfalfa and *Arabidopsis thaliana* was overexpressed in tobacco, the flower color faded or turned white. Transgenic tobacco flowers accumulated substances that turned DMACA blue. Recombinant *MtBAN* protein from prokaryotic expression catalyzed the conversion of anthocyanidins to EC. Based on the catalytic function described above, Xie and colleagues designated the protein encoded by the BAN gene as ANR. This was the first time that ANR had been proposed, and it clarified the previous misconception that the BAN gene was a homolog of LAR. For the first time, it was established that ANR plays a core role in the synthesis of proanthocyanidins by catalyzing the generation of ECs from anthocyanins (Liu et al. 2016). The catalytic mechanism proceeds through a highly reactive intermediate. Studies have identified an inherently labile catechin carbocation intermediate generated during the ANR‐catalyzed reaction (Wang et al. 2020). This intermediate can dynamically interconvert with EC‐type anthocyanidins in planta (Jun et al. 2021). Owing to its strong electrophilicity, it is readily captured by various nucleophiles—such as methanol, cysteine, and even catechin monomers—to form corresponding adducts (Wang et al. 2020).

Recent years have witnessed considerable progress in functional studies of ANR, particularly in the tea plant ( *Camellia sinensis* ), a species rich in catechins. When cyanidin and NADPH were added in vitro, the recombinant *CsANR* enzyme from tea plants produced (−)‐catechin and two chiral isomers of catechin, (+)‐EC and (−)‐EC (Pang et al. 2025). In vivo functional validation through transgenic approaches has further elucidated the physiological role of ANR. Overexpressing *CsANRα* from tea plants in tobacco led to the accumulation of proanthocyanidin‐like compounds. LC–MS analysis of transgenic tobacco phenolics showed a significant increase in catechin intermediates in *CsANRα‐overexpressing lines;* these intermediates are hypothesized to serve as extension units, acting as crucial precursors that drive proanthocyanidin polymerization (Wang et al. 2020). In 2016, Luo et al. investigated the function of *CsANR* by overexpressing it in tobacco and observed a significant accumulation of proanthocyanidin analogs (Luo et al. 2016). Notably, in *CsANRα* transgenic tobacco, there was a marked increase in catechin intermediates (Wang et al. 2020). These intermediates were hypothesized to serve as extension units for proanthocyanidin polymerization. Collectively, these studies demonstrate that in *Camellia sinensis* , CsANR catalyzes the conversion of anthocyanidins to EC‐type leucoanthocyanidins, which are subsequently reduced by LAR to yield EC. Concurrently, the highly reactive intermediates generated during ANR catalysis supply indispensable substrates for proanthocyanidin polymerization.

### 3.3 Anthocyanidin Synthase (ANS)

ANS is a key enzyme in the flavonoid metabolic pathway that catalyzes the conversion of leucoanthocyanidin to anthocyanidin. ANS is a member of the 2‐oxoglutarate/Fe (II)‐dependent dioxygenase (2‐OGDD) superfamily; its activity absolutely requires Fe^2+^, 2‐oxoglutarate and O_2_ as co‐substrates to catalyze the stereospecific oxidation of leucoanthocyanidins to the corresponding anthocyanidins (Wang et al. 2021). ANS has diverse functions: it can catalyze leucoanthocyanidin to anthocyanidin, act as flavonol synthase (FLS) to convert dihydroflavonol to flavonol and also catalyze (+)‐C to cyanidin and oxidized dimeric proanthocyanidin (Schilbert et al. 2021; Zhang et al. 2022). Researchers performed codon optimization and chemical synthesis on *CsANS*, *CsLAR*, and *CsANR*. The gene expression units were positioned between the 35S promoter and nopaline synthase terminator using a polyacrylamide gel electrophoresis (PAGE)‐mediated overlapping extension polymerase chain reaction method. The three genes were arranged in series for co‐expression using the same‐end enzyme technique. Then, the recombinant vector pYC1301 was transformed into tobacco. Two transgenic tobacco lines were ultimately obtained, showing vigorous growth. Twenty days after transplantation, the stems of transgenic tobacco showed preferential growth. This growth was more pronounced in 4‐week‐old transgenic tobacco, whose flowers also exhibited a deeper pink color. Moreover, the transgenic tobacco had significantly higher catechin content than the wild type. HPLC was used to measure catechin levels in wild‐type and two transgenic tobacco lines. Several catechin monomers (catechin, GCG, EC, ECG, EGC, and EGCG) were detected in the transgenic lines, with catechin present in the highest amount. In the transgenic tobacco, catechin averaged 10.44 mg/g DW, 4.4 times higher than the wild‐type's 2.38 mg/g DW. The EC, EGCG, and EGC levels also rose significantly by 57.8%, 51.9%, and 82.4%, respectively (Yang, Zhang, et al. 2024; Yang, Li, et al. 2024). In 2018, Jun et al. (Jun et al. 2018) distinguished the functions of *MtANS* and MtLDOX in alfalfa. They found that in alfalfa, the two have significantly different functions. The primary role of ANS in plants is anthocyanin biosynthesis, and LDOX mutation does not affect this process.

### 3.4 Catechin Gallate Transferase (ECGT)

From a structural perspective, the difference between ester‐type catechins and non‐ester‐type catechins lies primarily in whether the C ring undergoes galloylation. ECGT is precisely the enzyme that catalyzes the acyl transfer during the catechin acylation process. At the beginning of the 21st century, Li et al. purified a serine carboxypeptidase‐like acyltransferase (SCPL) from wild tomatoes (Li et al. 1999). Around the same time, Lehfeldt cloned an SCPL gene from an Arabidopsis mutant and expressed it as SMT, an apple acid acyltransferase. It was found that SMT uses the hydroxyl group of malic acid as a nucleophile to cleave ester bonds and catalyze acyl transfer. Currently, SCPL has been proven to catalyze the acylation of various substrates, such as terpenoids, flavonoids, and alkaloids. However, due to technical challenges in functional verification, most candidate genes for SCPL have not yet been functionally validated in most sequenced plants (Oda‐Yamamizo et al. 2023). Liu et al. (2012) purified ECGT from tea leaves and showed it could catalyze EGC and EC to form EGCG and ECG, respectively. To find the gene encoding ECGT, Yao et al. isolated the enzyme from tea leaves, obtaining two SCPL homologs, *CsSCPL4* and *CsSCPL5*. Results indicated *CsSCPL4* is a catalytic acyltransferase, while *CsSCPL5* is a non‐catalytic companion protein (NCCP). Co‐expression of these genes may cause galloylation, and their interaction enhances protein stability and promotes post‐translational processing (Yao et al. 2022). However, there are still some unexplained experimental results in the research, and further verification is needed for the genes related to ECGT.

### 3.5 UDP‐Glucose: Flavonoid‐3‐O‐Glucosyltransferase (UGGT)

UGGT, a UDPG‐dependent glycosyltransferase (UGT), can use flavonoid compounds as substrates and glycosylate them (Jiang et al. 2025). Glycosylation is one of the common biochemical reactions in plants, and it can alter the bioactivity, water solubility, stability, etc. of small molecules in plants. In 2008, Pang et al. (Pang et al. 2008) identified a gene encoding an EC‐specific glucosyltransferase, UGT72L1, in the seed coat of alfalfa, which can glycosylate the B‐ring 3′‐position of EC to form E3′G. They discussed the physiological significance of UGT72L1 and proposed the possible implications of flavan‐3‐ol B‐ring 3′‐position glycosylation: It may regulate favorable EC levels, serving as a potential detoxification mechanism or controlling the relative levels of initiating and extending units in proanthocyanidins (PA) synthesis. As an extending unit in PA synthesis, flavan‐3‐ol B‐ring 3′‐position glycosylation prevents possible oxidative polymerization and quinone formation at the 3′‐position, directing the correct 4–8 linkage between flavan‐3‐ol monomers, with the glycoside being removed after polymerization. The insertion of a UGT72L1 transposon in alfalfa seeds stabilizes or even enhances PA levels, supporting these proposals. Numerous experimental results currently indicate that gallic acid, isobutyric acid, sinapic acid, etc., can form organic acid glycosides under the action of UDPG glucosyltransferase. In pomegranate ( *Punica granatum* ), the UDP‐glucosyltransferase *PgUGT72BD1* specifically catalyzes the glucosylation of the 4‐OH position of gallic acid to produce gallic acid 4‐O‐glucoside (Chang et al. 2019). UGT72‐family enzymes (e.g., UGT72D1 and UGT72D7) catalyze the regiospecific 4‐O‐glucosylation of sinapic acid to yield sinapic acid 4‐O‐glucoside (Li, Wen, Lai, et al. 2024; Li, Zhang, Li, et al. 2024; Li, Borg, Krammer, et al. 2024; Li, Zhang, Cui, et al. 2024). Tea plants contain abundant glycosides, many of which are closely related to tea quality. They serve as precursors to aroma compounds and are linked to the glycosylation of phenolic substances. Flavonol glycosides are the most abundant glycosylated phenolic compounds (Shi et al. 2023).

So far, flavonoid synthesis has been a key focus in plant secondary metabolism research over the past decades. Catechin biosynthesis has been widely studied at biochemical and gene levels. The biosynthetic pathway of non‐esterified catechins in tea plants is relatively clear. However, there are still several scientific questions to be further explored regarding the synthesis, regulation and transport of catechin compounds in tea plants.

## 4 Tissue‐Specific Accumulation of Catechins in the Tea Plant

The specific accumulation of catechins in tea plants is synergistically regulated by a combination of internal and external factors (Table 3). Internal factors primarily involve the regulation of gene expression for key enzymes and transcription factors within the catechin biosynthesis pathway. Externally, environmental factors, such as the supply of nutrients like nitrogen and phosphorus, as well as other environmental influences, can significantly alter the synthesis and accumulation of catechins by impacting plant physiological metabolism. Consequently, breeding strategies and cultivation management practices must comprehensively consider these interconnected factors to optimize tea quality.

**TABLE 3:** Factors influencing the accumulation of catechins in tea plants.

Influencing factors	Influence the result	References
Lighting	Meanwhile, the response of gallic acid (GA) accumulation to light intensity was co‐regulated by genes associated with both photosynthesis and GA biosynthesis (CsaroB, CsaroDE1, CsaroDE2, CsaroDE3). Consequently, the epigallocatechin gallate content was enhanced due to the increased availability of its precursors (EGC and GA) and the upregulation of the CsSCPL gene	Xiang et al. (2021)
Lighting	Light intensity affected CsGLK1 and CsGLK2 transcript levels in tea plants, which in turn upregulates catechin biosynthesis, as evidenced by the elevated catechin content and related gene expression in CsGLK‐overexpressing tomato leaves	Wang et al. (2022)
Temperature	Catechin accumulation is driven by rainfall, temperature, and effective accumulated temperature (EAT) through the transcriptional regulation of key genes (CsCHS1, CsANR, CsSCPL). Warm, rainy environments induce EGCG, ECG, and TEC production via CsPAL and CsSCPL upregulation; in contrast, lower rainfall and EAT favor the accumulation of C, EGC, and TNEC	Tukhvatshin et al. (2025)
Geographical factors	The natural shading in hilly areas, resulting in reduced sunlight duration and intensity, led to higher accumulation of free amino acids in tea plants. In contrast, the open plain conditions favored the biosynthesis of total catechins (TC)	Wen et al. (2020)
Transcription factor	Nine R2R3‐MYB genes exhibited tissue‐specific expression in apical buds and young leaves, matching the accumulation sites of galloylated catechins. Three of these genes correlated strongly with biosynthetic genes, implicating them in the regulation of ECG and EGCG biosynthesis	Li et al. (2021)
Season	The accumulation of EGCG and ECG differed seasonally among cultivars: HJC1 and HJC2 had higher levels in summer, in contrast to HJC168, which peaked in spring	Huang et al. (2022)
Transcription factor	A combination of yeast one‐hybrid, dual‐luciferase, and EMSA assays revealed that CsWRKY12 activates CsSCPL4 and CsSCPL5 transcription by directly binding to W‐box elements in their promoters, thus upregulating key enzymes in galloylated catechin biosynthesis	Zhang et al. (2024)
Drought stress (DS)	Proposed mechanisms for catechin accumulation under DS involve direct ROS scavenging through proanthocyanidin polymerization, as well as indirect pathways including o‐quinone autogenesis and metal ion chelation paired with antioxidant system activation	Lv et al. (2021)
Nitrogen accumulation	The regulation of catechin accumulation and composition by nitrogen form was associated with changes in biosynthetic gene expression, wherein PAL, CHS, CHI, and DFR were upregulated under nitrate (${\mathrm{NO}}_{3}^{-}$) supply—with shoot DFR transcript levels showing a significant correlation with catechin content	Fan et al. (2015)
Phosphorus accumulation	Two transcription factors in the phosphate signaling pathway in tea regulate catechin biosynthesis by activating the transcription of CsANR1 and CsMYB5c, which further demonstrates that the Pi pathway inhibitor CsSPX1 can suppress the activation of CsANR1 and CsMYB5c by CsPHR1/2. The JA signaling inhibitor CsJAZ3 negatively regulates catechin biosynthesis through physical interactions with CsPHR1 and CsPHR2	Li, Wen, Lai, et al. (2024), Li, Zhang, Li, et al. (2024), Li, Borg, Krammer, et al. (2024) and Li, Zhang, Cui, et al. (2024)

Recent studies have demonstrated that there are significant differences in catechin monomers between tea plants ( *Camellia sinensis* ) and other species within the genus Camellia. In non‐tea group Camellia species, ester‐type catechins are nearly undetectable, primarily due to the low expression of the flavonoid 3′,5′‐hydroxylase gene (F3′5′H) (Liu et al. 2020; Jin et al. 2018). For instance, young leaves of Shucha Zao contain approximately 90 mg g^−1^ (dry weight) of EGCG, whereas Camellia tetracocca at the same developmental stage contains only 9 mg g^−1^ (dry weight) of EGCG. The absence of trihydroxylated catechins results in a total catechin content in fresh leaves of C. tetracocca that is only one‐third that of Shucha Zao (Jin et al. 2018). In addition to gene expression, the accumulation of catechins in tea plants of the same variety is also significantly influenced by the growing region. Compared to northern tea varieties, southern tea varieties have relatively higher contents of EC and ECG in fresh leaves (Jin et al. 2023).

Significant differences in the types and accumulation of catechins are observed across different developmental stages and organs of tea plants. In fresh leaves, monomeric catechins represent the predominant form of catechins. The eight monomeric catechins (catechin, GC, CG, GCG, EC, EGC, ECG, and EGCG) account for approximately 12%–24% of the dry weight of fresh leaves, with ester‐type catechins making up about 80% of the total catechins (Feng et al. 2024). Among fresh leaves at different developmental stages, the highest total catechin content is found in the first leaf, with a notable accumulation of ester‐type catechins. As leaves develop, the contents of ester‐type catechins ECG and EGCG significantly decrease, while the contents of non‐esterified catechins EC and EGC increase. Non‐esterified catechins typically reach their highest levels in the third to fourth leaves. This shift is primarily attributed to the reduced activity of ECGT and increased activity of tannase (*CsTA*) (Liu et al. 2012; Dai, Liu, et al. 2020; Dai, Lou, et al. 2020). In mature tea plants, both esterified and non‐esterified catechins significantly decline in older leaves.

In different tea plant tissues, monomeric catechins are most abundant in fresh leaves, followed by young stems, with the lowest content in roots. In the above‐ground parts of tea seedlings (leaves and shoot tips), monomeric and polymeric catechins, such as EC, EGC, and EGCG, accumulate, while in the below‐ground parts (roots and hypocotyls), B‐ring dihydroxylated catechins, such as proanthocyanidins (PAs), predominate. However, with the growth of the tea plant, the content in parts other than the root tips gradually decreases (Li, Wen, Lai, et al. 2024; Li, Zhang, Li, et al. 2024; Li, Borg, Krammer, et al. 2024; Li, Zhang, Cui, et al. 2024). In terms of monomeric catechin composition, fresh leaves exhibit the greatest diversity. The composition of catechin monomers in stems is similar to that in fresh leaves, while only EC is detected in roots. The main monomeric catechins accumulated in fresh leaves are EC‐EGC and EC‐EGCG. To date, 22 types of proanthocyanidins have been identified in fresh tea leaves by LC–MS (Zhuang et al. 2020). In contrast, the types of flavan‐3‐ols accumulated in tea plant roots are relatively limited, mainly consisting of EC‐type and EC‐type proanthocyanidins. In roots with low lignification, only small amounts of EC and C accumulate, while in highly lignified old roots, the main components are EC and EC‐type proanthocyanidins (Zhang et al. 2021).

Beyond the inherent tissue‐specific accumulation of catechins in tea plants, environmental factors also significantly influence catechin accumulation. Elucidating the regulatory mechanisms by which light, temperature, humidity, soil, and air affect the biosynthetic pathways of catechins in tea plants holds crucial theoretical and practical significance. Systematically dissecting the environment–metabolism interaction network not only provides a paradigm for understanding adaptive metabolic evolution in plants, but also offers key theoretical foundations and operational targets for optimizing tea quality and enhancing the content of characteristic functional components through ecological cultivation, precision agronomic regulation, and molecular breeding.

Temperature affects catechin composition through gene expression and regulatory enzyme activity. The SCP1 gene, which regulates galloylation, peaks in expression at 25°C; thus, ester‐type catechins (EGCG, ECG) accumulate the most between 12.7°C and 25.5°C. In contrast, non‐esterified catechins (EC, C) increase in content at low temperatures (< 15°C) because low temperatures inhibit UGT72A (the esterase gene) and activate ANR (Tukhvatshin et al. 2025). A higher diurnal temperature variation (25°C during the day/15°C at night) promotes catechin isomerization, increasing the conversion efficiency of EGC to EGCG by 12% (Tan, Li, et al. 2023; Tan, Vincken, et al. 2023). However, field trials demonstrated that exposure to temperatures above 35°C significantly down‐regulated the expression of catechin‐biosynthetic genes (e.g., *CsANS*, *CsLAR*), resulting in a 38%–50% reduction in total catechin accumulation. Modeling further confirmed that enzyme‐activity losses at > 35°C were positively correlated with the observed decline in catechin content (Tukhvatshin et al. 2025).

Water availability affects catechin metabolism through osmotic signaling and antioxidant responses. Moderate drought (soil water content at 40%–50%) activates the ABA signaling pathway, inducing the upregulation of LAR expression by MYB transcription factors, thereby promoting catechin accumulation (Tan, Li, et al. 2023; Tan, Vincken, et al. 2023). However, when soil water content falls below 30% under prolonged drought conditions, photosynthetic carbon fixation is inhibited, reducing the supply of catechin precursors. Simultaneously, the antioxidant system is triggered to consume catechins, leading to a decrease in total catechin content (Tan, Li, et al. 2023; Tan, Vincken, et al. 2023).

When atmospheric CO_2_ concentration and temperature rise concomitantly, the net accumulation of catechins is further amplified (Wang et al. 2021). Wang et al. (2024) directly examined the impact of elevated O_3_ on tea quality and demonstrated that ozone stress increases ROS accumulation, leading to a significant 15% reduction in total esterified catechins (including EGCG), whereas non‐ester forms remained largely unaffected. Within the catechin profile, concentrations of C, EC, and ECG declined markedly (by 22%, 31%, and 15%, respectively), while EGCG showed a downward trend that did not reach statistical significance (Wang et al. 2024). When the carbon‐to‐nitrogen ratio increases, carbon sources are preferentially allocated to secondary metabolites such as catechins (Hazra et al. 2021).

Soil factors primarily act indirectly. Increasing the solubility of aluminum ions (Al^3+^) to acidify the soil (pH 3.5–4.5) can significantly activate the expression of PAL and CHS genes, thereby promoting catechin synthesis. Experimental results indicate that the content of EGCG is the highest at pH 4.0 (Rigden et al. 2020). Low phosphorus stress induces the CsPHR1/2‐JAZ module, which, in concert with jasmonic acid, upregulates ANR, resulting in an 18% increase in catechin accumulation (Tukhvatshin et al. 2025). Excessively high rhizosphere temperatures may compromise membrane‐protein stability or perturb energy metabolism, thereby indirectly suppressing the activity of transport proteins such as MATE‐family efflux carriers and impeding the root‐to‐shoot translocation of catechins (Zhang et al. 2023).

## 5 Conclusions and Future Perspectives

**FIGURE 3:**
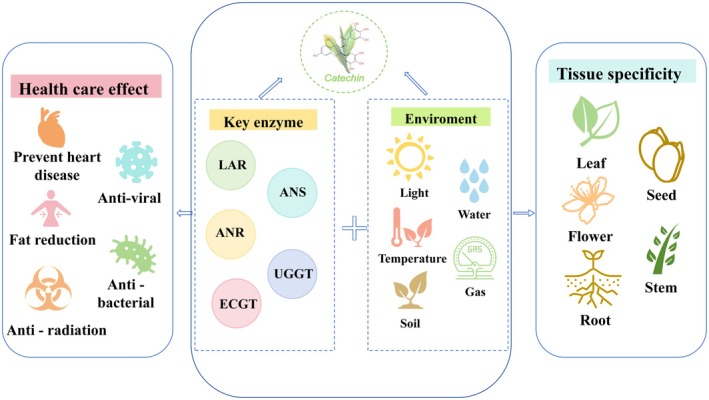
Factors, pharmacological effects, and accumulation of catechins in the biosynthetic pathway of tea plants.

Catechins are the primary secondary metabolites of tea plants and are crucial for tea quality and health benefits. This review summarizes the biosynthetic pathways and key enzymes of catechins in tea plants, the applications and synthetic methods of catechin derivatives, and the specific accumulation of catechins in tea plants (Figure 3). In biosynthesis, the downstream enzymes of catechin biosynthesis, including ANS, ANR, LAR, ECGT, and UGGT, not only determine the types of catechins in tea plants but also the final composition of catechins. Systematic elucidation of the synthetic mechanisms, precursor substances, and pharmacological actions of catechin derivative oxidation polymers, methylated catechins, and catechin‐theanine conjugates provides a theoretical basis for the efficient and green manufacturing and innovative applications of these highly active derivatives. The accumulation of catechins in tea plants exhibits distinct tissue specificity, a pattern closely related to the growth and development of tea plants as well as tea germplasm. Meanwhile, different environmental factors, by regulating the transcriptional network and enzyme activity, mediate the molecular mechanisms of catechin synthesis, which is the core scientific basis for optimizing tea quality and functional component content through precise environmental management.

Looking toward future progress, two pivotal avenues merit particular consideration. The first involves a deeper comprehension of catechin‐biosynthetic pathways and their associated enzymatic functions. When synergistically integrated with advanced plant nanotechnology and precise gene‐editing methodologies, this enhanced understanding offers significant promise for achieving targeted regulation of catechin content and composition within *Camellia sinensis* (Osmani et al. 2024). The second avenue addresses the intrinsic challenges posed by the inherent instability and limited bioavailability of catechins, necessitating the development of innovative application strategies. Nanotechnology‐based delivery platforms, with starch nanoparticles serving as a representative example, exhibit considerable potential for enhancing stability, enabling controlled release, and consequently expanding their utility across diverse fields such as fundamental plant science (Chen et al. 2025), functional food development (Chen et al. 2024), pharmaceutical applications (Chen et al. 2024), and other related sectors (Chen et al. 2024). Further dissection of catechin biosynthesis and function holds the potential to revolutionize the tea industry. It provides a foundation for quality‐oriented molecular breeding and precision agriculture, ultimately steering the sector toward a sustainable, resource‐efficient, and health‐driven future.

## Author Contributions

**Haiyi Yao:** data curation, writing – original draft. **Yuxin Gu:** data curation. Dibin Zhu: data curation, formal analysis. **Dibin Zhu:** data curation, formal analysis. **Dandan Tang:** investigation, validation, formal analysis. **Wei Chen:** investigation, validation, supervision. **Yongxian Chen:** methodology, funding acquisition, project administration, writing – review and editing. **Jiaojiao Zhang:** methodology, investigation, funding acquisition, project administration, writing – review and editing. **Liqiang Tan:** conceptualization, supervision, funding acquisition, methodology, project administration, writing – review and editing. All authors reviewed the manuscript.

## Funding

This work was supported by the Sichuan Innovation Team of National Modern Agricultural Industry System, sccxtd‐2024‐10, the Science and Technology Department of Sichuan Province, 2021YFYZ0025, the Ya'an City's “Open Call for Applications” Project, kczx2023–2025–01, the Scientific Research Foundation of Zhejiang A&F University, 2023CFR008, and the Suichang County–University Cooperative Science and Technology Program, SCX2025HZ24.

## Conflicts of Interest

The authors declare no conflicts of interest.

## Data Availability

The data supporting this study's findings are available from the corresponding author upon reasonable request.
